# Diffusion-weighted imaging for evaluating inflammatory activity in Crohn’s disease: comparison with histopathology, conventional MRI activity scores, and faecal calprotectin

**DOI:** 10.1007/s00261-016-0863-z

**Published:** 2016-08-27

**Authors:** D. A. Pendsé, J. C. Makanyanga, A. A. Plumb, G. Bhatnagar, D. Atkinson, Manuel Rodriguez-Justo, S. Halligan, S. A. Taylor

**Affiliations:** 1 University College London Hospitals NHS Foundation Trust, London, UK; 2 Centre for Medical Imaging, University College London, London, UK; 3 Research Department of Pathology, Cancer Institute, UCL, London, UK

**Keywords:** Crohns disease, Inflammatory bowel disease (IBD), Magnetic resonance imaging (MRI), Imaging, Diffusion

## Abstract

**Purpose:**

To evaluate whether the extent of enteric diffusion-weighted imaging (DWI) signal abnormality reflects inflammatory burden in Crohn’s disease (CD), and to compare qualitative and quantitative grading.

**Methods:**

69 CD patients (35 male, age 16–78) undergoing MR enterography with DWI (MRE-D) and the same-day faecal calprotectin (cohort 1) were supplemented by 29 patients (19 male, age 16–70) undergoing MRE-D and terminal ileal biopsy (cohort 2). Global (cohort 1) and terminal ileal (cohort 2) DWI signal was graded (0 to 3) by 2 radiologists and segmental apparent diffusion coefficient (ADC) calculated. Data were compared to calprotectin and a validated MRI activity score [MEGS] (cohort 1), and a histopathological activity score (eAIS) (cohort 2) using nonparametric testing and rank correlation.

**Results:**

Patients with normal (grades 0 and 1) DWI signal had lower calprotectin and MEGS than those with abnormal signal (grades 2 and 3) (160 vs. 492 μg/l, *p* = 0.0004, and 3.3 vs. 21, *p* < 0.0001), respectively. Calprotectin was lower if abnormal DWI affected <10 cm of small bowel compared to diffuse small and large bowel abnormality (236 vs. 571 μg, *p* = 0.009). The sensitivity and specificity for active disease (calprotectin > 120 μg/l) were 83% and 52%, respectively. There was a negative correlation between ileal MEGS and ADC (*r* = −0.41, *p* = 0.017). There was no significant difference in eAIS between qualitative DWI scores (*p* = 0.42). Mean ADC was not different in those with and without histological inflammation (2077 vs. 1622 × 10^−6^mm^2^/s, *p* = 0.10)

**Conclusions:**

Qualitative grading of DWI signal has utility in defining the burden of CD activity. Quantitative ADC measurements have poor discriminatory ability for segmental disease activity.

Magnetic resonance enterography (MRE) is now well established for the diagnosis and follow-up of Crohn’s disease [1–7], and there is considerable interest regarding its potential to quantify inflammation. Knowledge of inflammatory burden in Crohn’s disease is crucial to optimize treatment. In general, medication is effective at reducing inflammation but does not impact the long-standing fibrotic disease, which often necessitates surgery. Furthermore, in patients with established inflammation, monitoring therapeutic efficacy is necessary, so that drugs can be discontinued and/or replaced if ineffective. Several MRI activity scoring systems have been proposed and validated against various reference standards including histology, calprotectin, and colonoscopy [1, 8–10].

Recent data suggest that diffusion-weighted images (DWIs) may reflect biological activity [11–16]. Diffusion MRI generates image contrast contingent on the movement of water and other small molecules within tissue. Cellular infiltration associated with acute inflammation may alter DWI signal via restriction, so that image contrast may be related to disease activity.

Studies of DWI have used a variety of reference standards for inflammatory activity including barium fluoroscopy, endoscopy, surgical specimens, or even the MRI itself (raising the possibility of incorporation bias) [5, 9, 10, 13].

Furthermore, the majority of data supporting DWI have been obtained at the level of individual bowel segments (i.e., comparing measurements from a single segment such as the terminal ileum against a matched reference standard). In clinical practice, however, patient management is directed by the overall burden of inflammatory disease in the whole bowel, rather than from isolated segments. While DWI may be a rapid and accurate method to stage gastrointestinal inflammation overall, its utility as a global marker of activity has not been validated. It is also unclear whether simple qualitative grading of signal on DWI images is as effective as more time-consuming quantitative measurements (e.g., apparent diffusion coefficient [ADC] calculation).

The primary purpose of this study was to investigate whether qualitative evaluation of enteric DWI signal reflects overall inflammatory disease burden using two alternate standards of reference for global activity: faecal calprotectin and a validated MRI activity score. The benefit, if any, of adding DWI grading to the conventional MRI activity score to predict activity based on faecal calprotectin levels was also tested. The secondary purpose was to compare the sensitivity for segmental disease activity of qualitatively graded DWI images with calculated ADC measurements, when referenced against both histopathological and conventional MRI segmental activity scores.

## Methods

Regulatory and ethical approvals were obtained, and all prospectively recruited patients gave informed written consent.

### Study Population

A retrospective study of two separate patient cohorts was conducted (Table 1).

**Table 1:** Patient characteristics for Cohorts 1 and 2

Patient details	Cohort 1	Cohort 2
Total number	69	29
Male/female, n (%)	34/35 (49/51)	19/9 (66/34)
Median age at inclusion (years)	33	23
Previous intestinal resection, n (%)	30 (43)	2 (7)
Crohn’s disease phenotype (Montreal classification), n (%)		
A1	18 (26)	9 (31)
A2	43 (62)	16 (55)
A3	8 (12)	4 (14)
L1	14 (20)	5 (17)
L2	21 (30)	6 (21)
L3	43 (62)	18 (62)
L4	9 (13)	1 (3)
B1	31 (45)	24 (83)
B2	20 (29)	3 (10)
B3	20 (29)	2 (7)
*p*	19 (28)	1 (3)
Medication, n (%)		
No medication	6 (8)	19 (66)
Aminosalicylates/immunomodulators/steroids	44 (63)	7 (24)
Biological therapy	19 (28)	3 (10)

### Cohort 1

Consecutive adult patients with a histologically proven diagnosis of small-bowel or colonic Crohn’s disease were prospectively recruited from a study comparing inflammatory burden measured using a MRE activity score against the same-day faecal calprotectin. Patients were recruited from a single institution with an established secondary and tertiary inflammatory bowel disease service between Feb 2010 and Oct 2011. Part of the patient cohort and the recruitment protocol has previously been described [8].

### Cohort 2

A review of our institutional endoscopic database (from Nov 2013 to Nov 2014) was undertaken by the study coordinator (subspecialty-trained GI radiology Fellow with > 400 MRE case experience) to identify only patients fulfilling the eligibility criteria of (i) histologically proven diagnosis of Crohn’s disease, (ii) assessment with MRE including DWI, and (iii) endoscopic terminal ileal biopsy within 40 days (before or after) of MRI.

### MRI protocol

Magnetic resonance enterography (MRE) was performed using standard T2- and T1-weighted images (Table 2: MRI protocol). A 1.5T (Avanto; Siemens) and a 3.0T (Achieva; Philips) systems were used as per usual clinical practice at the recruiting institution. Patients were fasted for 4 h prior to drinking 1 to 1.5 l of 0.2% locust bean gum/2.5% mannitol solution at 45 minutes immediately prior to imaging [17]. Twenty milligrams of intravenous hyoscine butylbromide (Buscopan; Boehringer Ingelheim, Ingelheim, Germany) was administered together with 0.1 mmol/kg gadolinium (3 ml/s injection using a power injector).

**Table 2:** MR Enterography parameters: 1.5 T and 3.0 T

	Coronal/axial HASTE	Coronal/axial TrueFISP	Baseline VIBE	DCE VIBE
MRE parameters: 1.5 T				
FOV (mm)	Variable	Variable	Variable	Variable
No. slices	20/26	25/34	40	40
Stacks	1/3	1/3	1	1
Repetition time (ms)	1200/800	3.98/4.25	3.07	2.73
Echo time (ms)	86/86	1.72/2.13	1.08	0.9
Image matrix	256/256	256/256	256	256
Slice (mm)	4/4	4/4	3.5	3.5
Averages	1	1	1	1
Flip angle			15°	15°
MRE Parameters: 3.0T				
FOV (mm)	Variable	Variable	Variable	Variable
No. slices	34/69	36/37	82	80
Stacks	1/1	1/1	1	1
Repetition time (ms)	1200/1100	2.5/3.3	2.3	2.3
Echo time (ms)	80/80	1.24/1.66	1.13	1.04
Image matrix	528/512	400/240	576	224
Slice (mm)	4/4	5/5	2	2
Averages	1	1	1	1
Flip angle	90°/90°	45°/60°	10°	10°

Dynamic contrast-enhanced VIBE commenced at the start of contrast injection and the final time point was used to assess contrast enhancement

*HASTE*, Half-fourier acquisition single-shot turbo spin echo; *TrueFISP*, True fast imaging with steady state precision; *VIBE*, volumetric interpolated breath hold examination

Prior to contrast-enhanced imaging, DWI sequences were also acquired. Specifically, free-breathing axial diffusion MRI was performed using an echo-planar imaging (EPI) sequence, and spectrally adiabatic inversion recovery (SPAIR) was applied for fat-suppression. Six b-values were obtained (0, 50, 100, 300, 600, 800 mm^2^/s) at 3.0T and 5 (0, 50, 100, 300, 600 mm^2^/s) at 1.5T. 4 signal averages were taken for each b-value.

### Analysis

#### Qualitative grading of enteric DWI for evaluating global disease inflammatory activity

##### Qualitative global DWI grading

Visual assessment of DWI data from all patients in cohort 1 was performed in consensus by 2 study readers, sub-specialist GI radiologists (with 3 and 5 years of experience).

DWI images and ADC maps alone (with no anatomical images) were uploaded on Osirix, [an open-source DICOM viewer www.osirix-viewer.com ] workstation for analysis. Readers were blinded to the standard MR enterography images and to all clinical data but were aware of the purpose of the study.

Using all available B values, readers in consensus graded the mural signal intensity of the small bowel as a whole, and the colon on a 4-point scale (Grade 0—Normal, 1—Probably Normal, 2—Probably Abnormal, 3—Abnormal) with reference to normal bowel, using the method described by Oto et al [18]. If there were differing grades of diffusion abnormality within the small bowel or colon, the highest grade was recorded. In addition, using electronic calipers, readers measured the longitudinal extent of abnormal (grade 2 or 3) small-bowel signal (<10 or ≥10 cm).

**Fig. 1:**
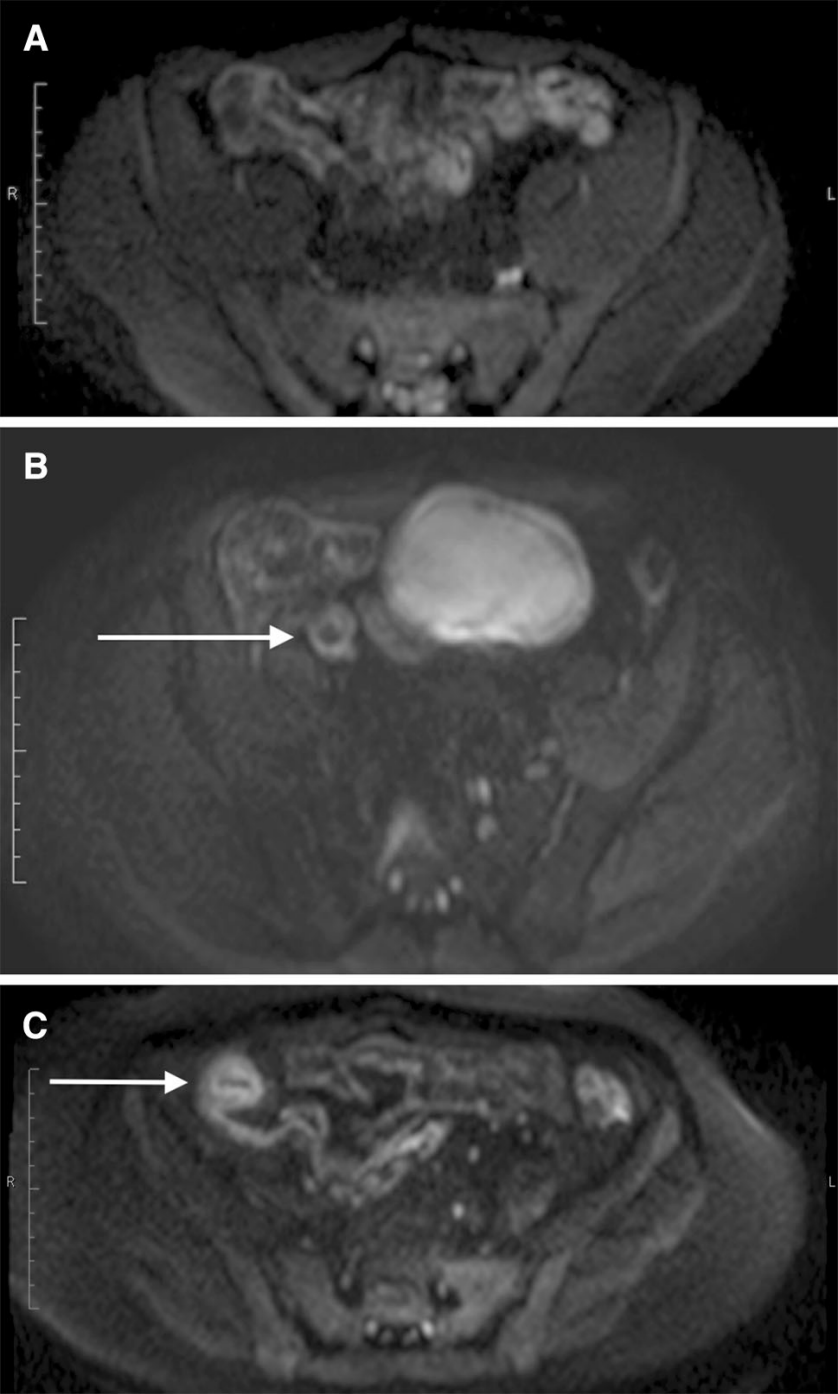
Example diffusion images demonstrating scoring schema. **A** Probably Normal (grade 1). *B* = 600 image of small bowel showing pelvic ileal loops with probably normal (grade 1) DWI signal. **B** Probably Abnormal (grade 2): *B* = 600 image of small bowel showing moderately high signal ileum (*arrow*). **C** Definitely Abnormal (grade 3): *B* = 600 image of small-bowel very high-signal terminal ileum (*arrow*)

Examples of the grading system are shown in figure 1.

##### Reference standards-global disease activity

- *Calprotectin* All patients in cohort 1 provided a stool sample on the day of MRI examination, which was used for the same-day measurement of faecal calprotectin (fC) (PhiCal; NovaTec Immunodiagnostica, Dietzenbach, Germany)
- *Global MRI activity score* A previously validated global MRI activity score, the MRE global score [MEGS], was applied to all datasets in cohort 1 as part of a previously published study [8]. This was performed by the study coordinator in consensus with an experienced subspecialty-trained gastroenterologist (2 years of experience of MR enterography). In brief, a score is assigned to each of 9 segments (rectum, sigmoid, descending, transverse, ascending, caecum, terminal ileum, ileum, and jejunum) based on qualitative scoring (0–3) of mural thickness T2 signal, mural enhancement and pattern, peri mural T2 signal, and colonic haustral loss. Segmental scores are then multiplied by 1 if segmental disease length is 1 to 5 cm, 2 if > 5 < 15 cm, and 3 if >15 cm, and a score of 5 then added for the presence of abscess, fistula, comb sign or lymphadenopathy

#### Comparison of qualitatively graded DWI signal with calculated ADC measurements for evaluating segmental disease activity

##### Qualitative segmental DWI evaluation

Both readers in consensus qualitatively graded DWI signal in the last 5 cm of the terminal ileum in all patients in cohort 2, again using the criteria of Oto et al. [18], described above. Readers were blinded to the standard MR enterography images and to all clinical data and utilized DWI images and ADC maps alone, uploaded on Osirix.

##### Quantitative segmental DWI evaluation

The study coordinator reviewed the MRI datasets in patient cohort 1 and identified only those with unequivocal ileal disease on conventional MRI sequences using standard criteria [19]. After a wash out period of 1 month following the qualitative grading, the 2 readers reviewed the DWI images of the selected subset of cohort 1 and all cohort 2 datasets blinded to clinical data, conventional MRE sequences, and ADC maps. Quantitative analysis of the DWI data was performed independently by each of the readers. Specifically, regions of interest (ROIs) were placed using OsiriX on the DWI images in the wall of the most abnormal area of ileum (cohort 1) or terminal ilium with 5 cm of the ileocecal valve (cohort 2). The ROI was placed initially on the highest *B*-value image (*B* = 600 mm^2^/s (1.5T) or *B* = 800 mm^2^/s (3T). In order to mitigate against bowel wall movement, the regions of interest were automatically propagated throughout the DWI dataset by the software and then manually readjusted such that they remained anatomically constant.

The conventional monoexponential diffusion model *S* *=* *S*_*0*_.exp(−*b*.ADC) was fitted to the data to estimate a single diffusion coefficient, ADC from each ROI. The mean ADC for the two readers was calculated for each patient and used for subsequent analysis.

##### Reference standards-Segmental disease activity

- Histopathology activity score. In each patient from cohort 2, the terminal ileal biopsies were stained with haematoxylin–eosin and retrospectively reviewed by an experienced pathologist >10 years of experience, who was unaware of clinical information or MRI findings. The histopathologist applied an endoscopic biopsy acute inflammatory score (eAIS: Table 3) based on the typical morphological features of Crohn’s disease described in guidelines published by the European Crohn’s and Colitis Organization [20] first proposed by Steward et al [1]. At least three samples of terminal ileum biopsy were collected for each patient, and the highest score for each was used for that patient, in accordance with the standard procedure in our institution.

**Table 3:** Histopathology grading for acute inflammation score (AIS)

Histological variable	Grade
Erosion or ulceration	0 = No, 1 = Yes
Polymorphs in the lamina propria	0 = No, 1 = Yes
Cryptitis	0 = No, 1 = Yes
Crypt abscess formation	0 = No, 1 = Yes
Inflammatory exudates	0 = No, 1 = Yes
Granulomas	0 = No, 1 = Yes
- Conventional MRI activity score. For the subset of patients from Cohort 1 with ileal disease, the MEGS scores for the ileum and terminal ileum were summed to give a combined total ileal score (iMEGS). A cut-off of >10 points was used to define active disease based on the previous MEGS validation work, in which a score of <10 indicated a segment with little or no disease activity (equivalent to a score of 0 or 1 for each individual parameter comprising the score) [8].

### Statistical methods

Statistical analysis was performed using JMP v10 (SAS Institute Inc., Cary, NC, USA). Shapiro–Wilk *W* test was used to evaluate for normal distribution of data.

The primary set of analysis assessed the qualitative grading of enteric DWI signal to evaluate global inflammatory disease burden.

Specifically, all cohort 1 patients were grouped into either grade-0 and grade-1 (normal) or grade-2 and grade-3 (abnormal) DWI abnormality and calprotectin level compared between the two groups using the Mann–Whitney *U* test. The analysis was repeated using MEGS, as the second global reference standard.

Thereafter, in those with abnormal (grade 2 and 3) DWI, calprotectin levels were compared across those with abnormality in (i) <10 cm of small bowel, (ii) ≥10 cm of small bowel, (iii) both small bowel and colon, and (iv) colonic only using Kruskal–Wallis test with post hoc correction.

Finally, the theoretical diagnostic benefit of adding DWI grading to conventional MEGS in predicting disease activity based on the calprotectin reference alone was evaluated. Patients in cohort 1 were divided into two groups using a previously proposed calprotectin cut-off of ≥120 μg/l for active disease (vs. <120 μg/l for nonactive disease) [21]. The sensitivity and specificity of DWI alone (taking grade 2 or 3 to be active), MEGS alone (taking >10 points to be active), and combined MEGS plus DWI for active disease were calculated.

The secondary set of analysis evaluated qualitatively grading of DWI images and calculated ADC measurements for assessing segmental disease activity.

Bland–Altman analysis was used to test agreement between all ADC measurements in both study cohorts made by the 2 readers. The mean of the 2 readers’ measurements was used in subsequent analysis.

Using the histopathological standard of reference (cohort 2), ADC values were compared between patients with no histological inflammation (eAIS = 0) and those with histopathological inflammation (AIS score ≥1) using the Mann–Whitney *U* test. eAIS scores were then compared across qualitative DWI grades using Kruskal–Wallis test with post hoc correction. The sensitivity and specificity of DWI grades 2 and 3 for active disease (AIS >1) were calculated.

Using the conventional MRI activity score as the standard of reference (cohort 1 with unequivocal ileal disease), the correlation between ADC and iMEGS was tested using Pearson rank correlation. Thereafter, patients were grouped into either active or nonactive segmental disease based on an iMEGS of >10 points, and ADC compared between the two groups using the Mann–Whitney *U* test.

The accuracy of ADC in predicting the presence of inflammation on biopsy (eAIS >0) was quantified by calculating the area under the receiver operating characteristic (ROC) curve.

## Results

### Patient demographics

There were 71 patients (35 male, age 16–78) in cohort 1. Two patients were excluded due to the absence of DWI data leaving 69 patients.

Cohort 2 consisted of 29 patients (19 male, age 16–70). The mean time between colonoscopy and biopsy and MRI was 2 days (range 0–37 days).

Baseline patient characteristics are shown in Table 1.

#### Qualitative grading of enteric DWI for evaluating global disease inflammatory activity

##### Qualitative global DWI grading

20 of 69 patients (29%) were evaluated as having grade 1 (probably normal) DWI on visual assessment, 17 (25%) grade 2 (probably abnormal), and 32 (46%) grade 3 (definitely abnormal) DWI. No patients were assigned grade 0.

Based on the extent of abnormal DWI (grade 2 and 3), 30 patients had <10 cm of small-bowel abnormality, 15 ≥ 10 cm of small-bowel abnormality, 11 small-bowel and colon abnormality, and 13 colonic abnormality only.

##### Calprotectin Reference standard

Mean calprotectin (fC) was 398 μg/g (range 0–1970). The fC was increased (>120 μg/g) in 44/69 (64%).

**Fig. 2:**
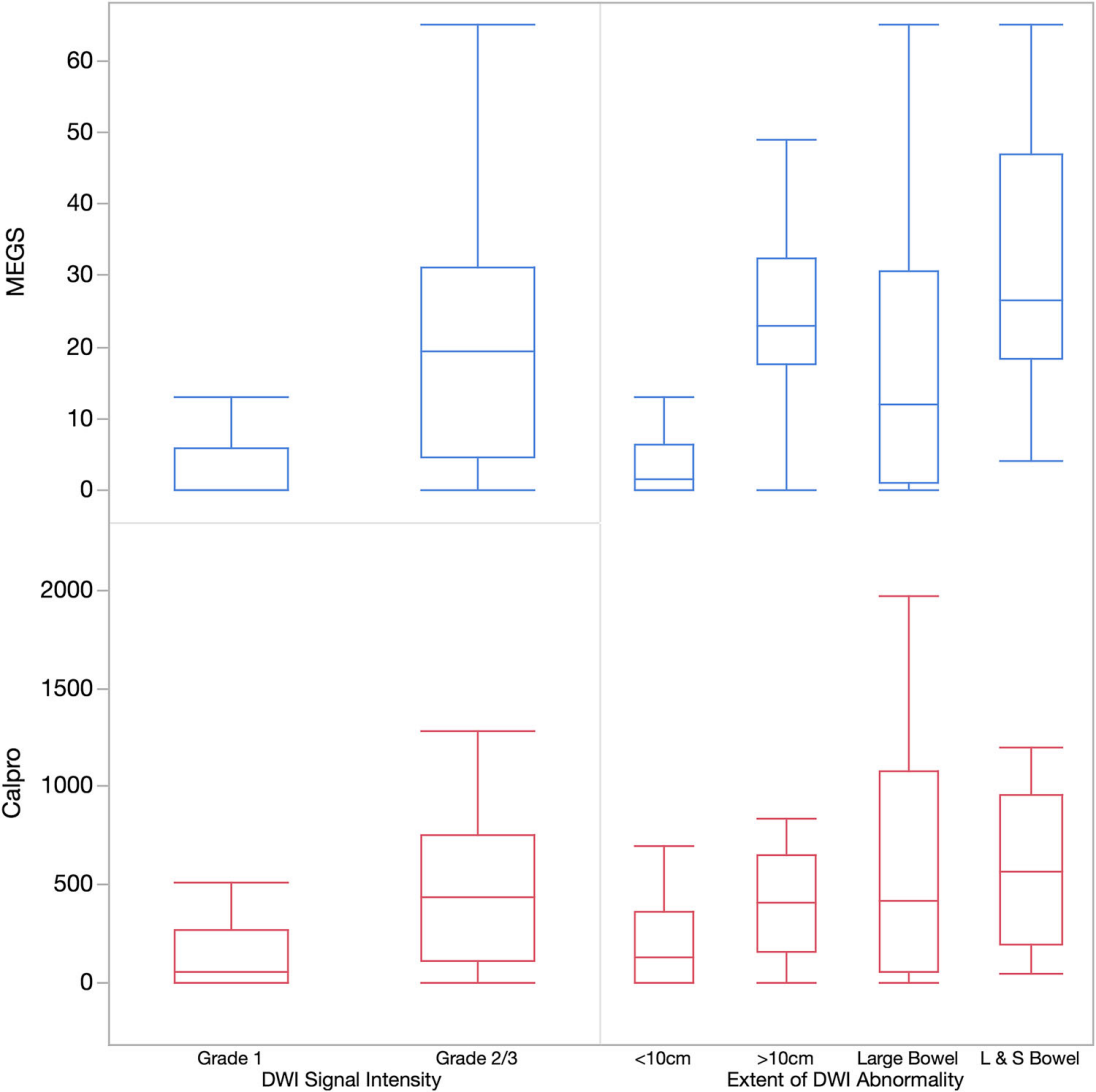
Boxplots illustrating qualitative DWI grading against faecal Calprotectin and MEGS (median values, quartiles, and range)

Patients with normal (grade 1) DWI had significantly lower mean calprotectin level than those with abnormal (grades 2 and 3) DWI (160 ± standard deviation (SD) 257 μg/l vs. 492 ± SD 422 μg/l) (*p* = 0.0004) (Figure 2).

Calprotectin levels were significantly lower in those with abnormal DWI limited to <10 cm of small-bowel compared to those with abnormal DWI involving both the small and large bowel [(236 ± SD 302 μg/l vs. 571 ± SD 390 μg/l) (*p* = 0.009 assuming an adjusted alpha-level of 0.0125] (Fig. 2)

##### Global MRI activity score (MEGS) reference standard

Mean MEGS was 15.8 (range 0–65). MEGS was increased (> 10) in 35/69 (51%)

When using MEGS as the standard of reference, patients with normal DWI grades had significantly lower MEGS than those with abnormal DWI grades (3.3 ± SD 5.5 vs. 21 ± SD 18) (p < 0.0001) (Figure 2).

##### Diagnostic performance of DWI for detecting active disease

The sensitivity and specificity of abnormal DWI (grades 2 and 3) for detecting biochemically active disease (defined by calprotectin > 120 μg/l) were 83% (95% CI 69% to 92%) and 52% (95% CI 31% to 73%), respectively, whereas for MEGS alone they were 88% (73% to 97%) and 54% (95% CI 37% to 71%). Combining DWI with MEGS produced marginal benefit with sensitivity of 91% (95% CI 76% to 98%) and specificity 56% (95% CI 38% to 72%).

#### Comparison of qualitatively graded DWI signal with calculated ADC measurements for evaluating segmental disease activity

##### Quantitative DWI evaluation

There was no significant difference in the mean ADC between those measured with 1.5T (1679 ± SD 496 × 10^−6^mm^2^/s) and 3.0T (1596 ± SD 414 × 10^−6^mm^2^/s) scanners (*p* = 0.6).

Mean ROI size for all ADC measurements was 0.81 ± SD 0.65 cm^2^ for observer 1 and 0.72 ± SD 0.58 cm^2^ for observer 2.

**Fig. 3:**
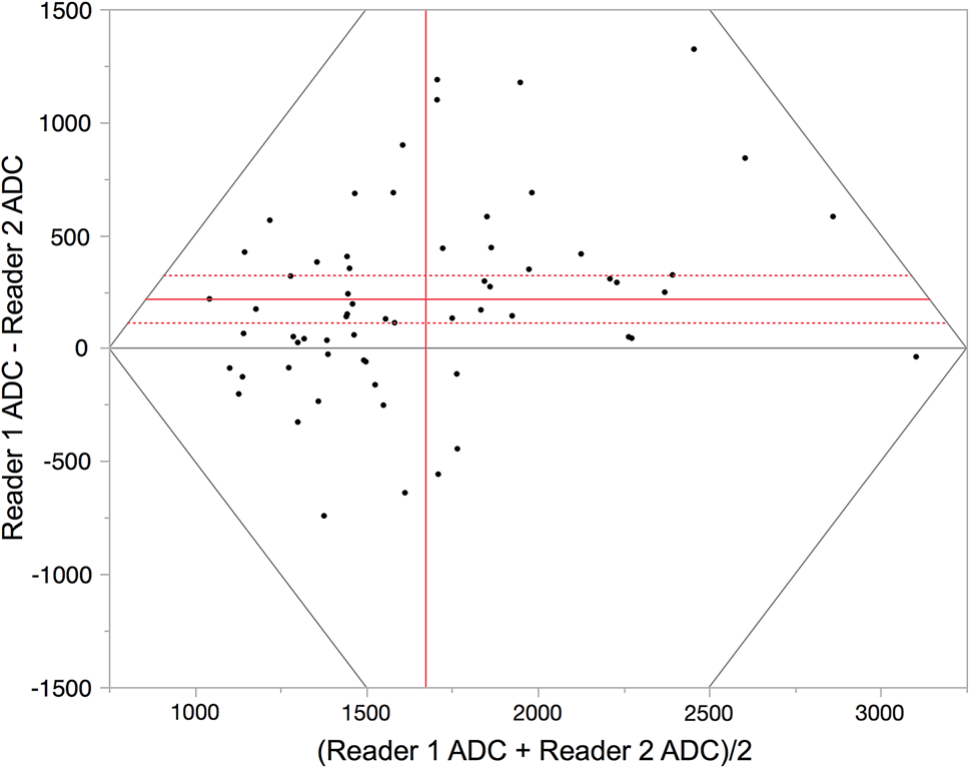
Bland–Altman plot illustrating reader Agreement for ileal ADC measurement

A Bland–Altman plot of reader agreement for all ADC measurements is shown in figure 3. The 95% limits of agreement were +52 and +329 × 10^−6^ mm^2^/s, with a mean difference of 226 × 10^−6^ mm^2^/s.

#### Conventional MRI activity score reference standard

33 patients (17 male, age 18–62) in cohort 1 had unequivocal ileal disease based on conventional MRE criteria. Overall, 17/33 (52%) had nonactive disease (iMEGS < 10) and 16/33 (48%) had active disease (iMEGS > 10).

There was a moderate negative correlation between iMEGS and mean ADC across the 2 readers (*r* = −0.41, *p* = 0.017)

**Fig. 4:**
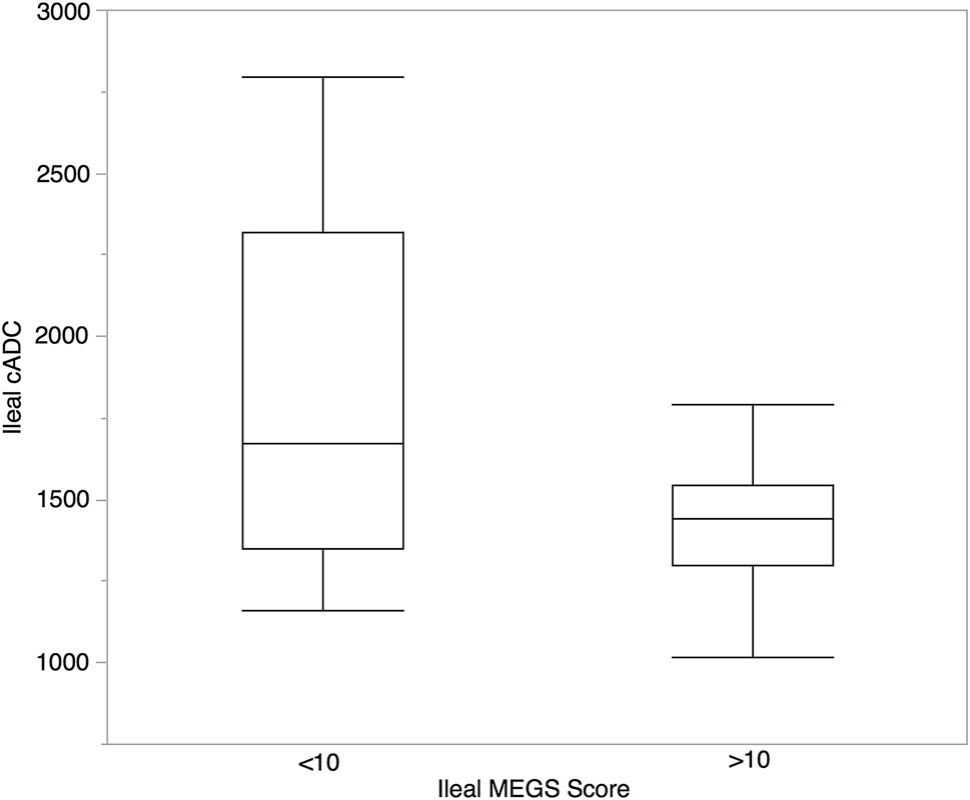
Boxplots illustrating calculated ileal ADC (mean of 2 readers) in patients with nonactive disease (iMEGS < 10) and active disease (iMEGS > 10). Boxplots showing median values, quartiles and range. There was no significant difference in cADC between the groups *p* = 0.10

Mean ADC in patients with inactive disease based on conventional MRI was not significantly different than those with active disease (1801 ± 537 × 10^−6^ mm^2^/s vs. 1473 ± 278 × 10^−6^ mm^2^/s) p = 0.10 (Fig. 4).

##### Histopathology activity score reference standard

Histological eAIS scores from cohort 2 patients ranged from 0 to 4. Seven patients had an eAIS score of 0, and inflammation (AIS > 1) was present in 22/29 (76%).

#### Qualitative DWI

For qualitative grading of the terminal ileal DWI signal, 6 patients (21%) were assigned grade 1, 9 (31%) grade 2, and 14 (48%) grade 3.

There was no significant difference in eAIS between qualitative DWI scores (*p* = 0.42).

The sensitivity and specificity of abnormal (grades 2 and 3) diffusion score for histological activity (eAIS > 1) were 82% (95% CI 60% to 95%) and 29% (95% CI 4% to 71%).

#### Quantitative DWI

**Fig. 5:**
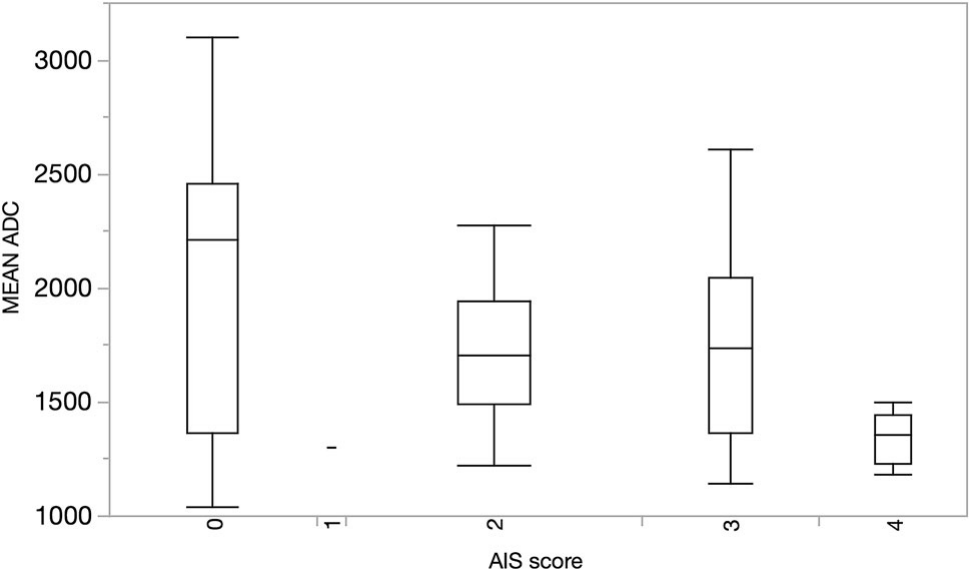
Boxplots illustrating ADC (median values, quartiles, and range) grouped by the histological eAIS score

The mean ADC in those without inflammation was not significantly different from patients with inflammation (2077 ± SD 696 × 10^−6^ mm^2^/s vs. 1622 ± SD 367 respectively) (*p* = 0.10) (Fig. 5)

ROC analysis of ADC as predictor of the presence (AIS > 0) or absence (AIS = 0) of pathological inflammation on biopsy had an area under the curve (AUC) of 0.71 with sensitivity of 86% and specificity of 58% at ADC = 1926 × 10^−6^ mm^2^/s.

## Discussion

This study aimed to evaluate the accuracy of subjective grading of DWI signal and objective measurement of ADC in grading of Crohn’s disease activity using a range of segmental and global standards of reference.

Our results were variable, with some data supporting the use of qualitative rather than quantitative DWI grading, but little evidence of utility of either against a segmental histopathological reference.

Although assessment of disease activity is vital in management of Crohn’s disease, there is no single accepted standard of reference with clinical scores, endoscopic, pathological, and biochemical markers used in clinical practice. In the present study, we used 3 reference standards: (1) faecal calprotectin, a biochemical marker of global gut inflammation; (2) a validated MRI global and segmental activity score [8]; and (3) histopathological analysis of endoscopic terminal ileal biopsy.

We found faecal calprotectin levels were significantly greater (reflecting greater disease activity) when enteric DWI was abnormal. The findings were similar when using MEGS as the standard of reference. The presence of any abnormal DWI signal had 83% sensitivity for active disease based on faecal calprotectin. However, specificity was very low at just 52%. We tested the theoretical addition of DWI to conventional MRI activity grading using MEGS and found a marginal benefit in terms of detecting active disease based on the calprotectin reference (increasing sensitivity from 88% to 91%). Our data are broadly similar to that of Kim et al, who also reported a small improvement in sensitivity for bowel inflammation by adding DWI to conventional sequences, at the expense of reduced specificity [22]. These data suggest DWI cannot replace conventional imaging sequences and is at best an adjunct to be used in combination. Although recent data suggest DWI may be able to replace contrast-enhanced imaging in standard protocols [5], it is clear that conventional MRI sequences are vital in maintaining specificity.

We did however find evidence that the longitudinal extent of DWI abnormality in the small bowel and colon was related to calprotectin level, i.e., in general, the more widespread the DWI abnormality, the greater the calprotectin level and inflammatory disease burden. A rapid review of DWI images noting the severity and extent of abnormal DWI signal may therefore provide a convenient and efficient method of assessing overall activity in patients with already known disease on conventional sequences. Such an approach could be useful in assessing treatment effect by rapidly comparing pre- and post-DWI imaging, for example.

To our knowledge, this is the first work to compare DWI with calprotectin. One advantage of calprotectin is that it better reflects total inflammatory burden than segmental references such as endoscopy which can assess at most the colon and terminal ileum. It does have limitations however. It may better reflect colonic rather than small-bowel Crohn’s disease [23], and levels are not necessarily linearly related to activity. These levels may also not be increased in the presence of active disease, or conversely may be falsely increased in the absence of inflammation [24]. Many workers have therefore compared DWI with conventional MRI activity scores, which we also explored in the present study. Hordonneau et al, for example, found the subjective presence of abnormal DWI had high sensitivity for segmental disease activity defined by a Maria score >7 [13]. Our data also suggested a relationship between DWI and an MRI activity score [8], although not as strong as those reported by Hordonneau et al. An important strength of the present study is that readers were fully blinded to conventional MRI sequences when grading DWI signal. Such an approach better isolates the true diagnostic worth of DWI and limits the major risk of incorporation bias when MRI is used as a standard of reference for new sequences.

Our data regarding the utility of quantitative ADC measurement was relatively poor. Similar to previous work [13], we did find a negative correlation between ADC and a segmental MRI activity score, although the strength of this association was weak.

Against a histopathological score of activity, neither qualitative segmental DWI grades nor ADC measurement showed a statistically significant relationship to the level of inflammation. This is at odds to previous studies which have shown reasonable correlations between both ADC and subjective DWI grading and an endoscopic standard of reference [16, 22].

Part of the discrepancy could in part be due to the standard of reference employed. The histopathological score is a based on mucosal biopsy whereas ADC measurements are taken from the whole bowel wall. The pathological scoring system has however been employed with success in other imaging studies [1, 25]. Furthermore, endoscopic standards of reference also suffer from the same limitation of assessing only the bowel mucosa and not the full transmural disease extent. Of note, in a detailed imaging–pathological correlation study using surgical resection specimens, Tielbeck et al found no association between ADC and a full wall thickness histological score of inflammation [26] which concurs with our findings.

Quantitative DWI analysis, with calculation of ADC, is a complex task. Several post-processing steps are necessary. First of all, regions of interest must be placed on the diffusion image sequences. These are then either transferred to the post-processed ADC maps for ADC calculation, or (as was performed in this study) the ROI is propagated through the each DWI sequence and the ADC values calculated directly.

The measurement of ADC in bowel is often difficult due to the relatively thin bowel wall, given the typical image slice thickness (normal bowel wall 1–2 mm, typical DWI slice thickness 5–8 mm). Bowel peristalsis during image acquisition introduces further error. These errors can be limited by shortening image acquisition time (e.g., fewer signal averages), but at the expense of a reduction in signal to noise. It is perhaps not surprising that interobserver agreement for measurement of ADC was poor between 2 readers in the present study. Indeed, it could be argued that the apparent reported reduction in ADC in active disease is in part due to overestimating ADC in the thin wall of normal bowel or that affected by fibrotic disease. Wall thickness per se is a strong predictor of activity [8–10, 27], and measurement of ADC is easier in thickened bowel.

There are several limitations in this study. We used both prospectively recruited and retrospectively identified cohorts, although this was deliberate given our wish to apply a variety of reference standards. Patient numbers in each group were reasonable but not informed by any power calculation. As noted above, there is no single accepted reference standard for activity. By using calprotectin, an MRI activity score, and histopathology, we attempted to capture the strengths of several alternatives but did not employ others such as endoscopy grading or surgical specimens. We acquired data on 1.5 T and 3 T MRI, although we found no difference in ADC measurements between these two platforms. As noted above, a strength of our methodology is that we blinded readers to all conventional sequences when evaluating DWI to truly isolate its diagnostic potential. In clinical practice, this is not how DWI is employed. Similarly, the study design utilized consensus reading of DWI data, which may limit the generalizability of these results. The MR protocol was originally designed to allow biexponential quantification of diffusion data and therefore used many B-factors. Although the IVIM model has been shown to be of value [12], this protocol increases scan time significantly and therefore is prone to image degradation from bowel movement.

In conclusion, qualitative (visual inspection) assessment of DWI is a reasonably sensitive tool for detection of bowel inflammation in Crohn’s disease based on a calprotectin (but not histopathological) standard of reference but has poor specificity and cannot fully replace conventional sequences. Its ability to grade the severity of inflammation is however limited. In those with known disease, rapid review of DWI could give a reasonable estimate of the overall active disease burden. The addition of DWI to conventional MR enterography sequences has a marginal benefit in increasing diagnostic accuracy. Quantitative ADC measurements are prone to poor interobserver agreement, have lower discriminatory ability for active disease, and are therefore not recommended.
